# One Health Approach to Study the Occurrence and Antimicrobial Resistance of Extended-Spectrum β-Lactamase- and Carbapenemase-Producing *Escherichia coli* and *Klebsiella* spp. in Urban Agriculture in Burkina Faso

**DOI:** 10.3390/microorganisms12112170

**Published:** 2024-10-29

**Authors:** Fatimata Bintou Josiane Diarra, Isidore Juste Ouindgueta Bonkoungou, Zakaria Garba, Namwin Siourimè Somda, Djifahamaï Soma, Marguerite Edith Malatala Nikiema, Evariste Bako, Souleymane Sore, Natéwindé Sawadogo, Nicolas Barro, Kaisa Haukka

**Affiliations:** 1 Department of Biochemistry and Microbiology, Université Joseph KI-ZERBO, Ouagadougou 03 BP 7021, Burkina Faso; josiane.diarra@ujkz.bf (F.B.J.D.); isidore.bonkoungou@ujkz.bf (I.J.O.B.); somadjifa@ujkz.bf (D.S.); nicolas.barro@ujkz.bf (N.B.); 2 Clinical Research Unit of Nanoro, Institut de Recherche en Sciences de la Santé, Ouagadougou 11 BP 218, Burkina Faso; zakaria.garba@crun.bf; 3 Département Technologie Alimentaire (DTA)/IRSAT/CNRST, Ouagadougou 03 BP 7047, Burkina Faso; namwin.somda@cnrst.gov.bf; 4 Laboratoire de Virologie et Biotechnologies Végétales, Institut de L’Environnement et de Recherches Agricoles (INERA), CNRST, Ouagadougou 04 BP 8645, Burkina Faso; marguerite.nikiema@inera.bf; 5 Department of Biochemistry and Microbiology, Centre Universitaire de Tenkodogo, Ouagadougou 12 BP 417, Burkina Faso; evaristebako@uts.bf; 6 Direction des Laboratoires de Biologie Médicale, Ministère de la Santé, Ouagadougou 03 BP 7022, Burkina Faso; soulsore11@yahoo.fr; 7 Department of Sociology, Université Thomas SANKARA, Ouagadougou 12 BP 417, Burkina Faso; natewinde.sawadogo@ujkz.bf; 8 Department of Microbiology, University of Helsinki, 00014 Helsinki, Finland

**Keywords:** *Escherichia coli*, *Klebsiella* spp., ESBL, market gardens, Burkina Faso

## Abstract

Data on antimicrobial resistance in Burkina Faso’s agricultural sector is still limited. This study assessed the occurrence of extended-spectrum β-lactamase-producing *Escherichia coli* (ESBL-*Ec*) and *Klebsiella* spp. (ESBL-*K*) in lettuce, environment, and gardeners’ stools in market gardens in Ouagadougou, Burkina Faso. A total of 356 samples were collected from three vegetable gardens (76 lettuce, 76 soil, 62 manure, 63 irrigation water, and 79 human stools). The ESBL-selective medium was used for initial selection of ESBL-producing bacteria, and the isolates were further identified using biochemical tests. An antibiotic susceptibility test was performed using the disk diffusion method. The overall prevalence of ESBL-*Ec* and/or ESBL-*K* in the samples was 232/356 (65.2%). Of the lettuce samples, the prevalence of ESBL-*Ec* was 19/76 (25.0%) and ESBL-*K* 33/76 (43.4%). In the market gardens environment, the prevalence of ESBL-*Ec* was 32/201 (15.9%) and ESBL-*K* 124/201 (61.7%). In the gardeners’ stools, the prevalence of ESBL-*Ec* was 42/79 (53.2%) and ESBL-*K* 24/79 (30.4%). Two ESBL-*K* isolates were found to produce NDM carbapenemase. Due to the high prevalence of ESBL-producing bacteria, which may also be carbapenemase producers, it is necessary to monitor pathogens in agricultural products with a “One Health” approach to limit and prevent infections in the population.

## 1. Introduction

The spread of multidrug-resistant (MDR) bacteria is a major problem affecting public health globally. In 2019, about 4.95 million deaths were associated with bacterial antimicrobial resistance (AMR) worldwide, with 1.27 million deaths directly attributable to MDR bacteria [1]. In Western sub-Saharan Africa, 27.3 deaths per 100,000 attributable to AMR were reported in 2019 [1]. AMR substantially reduces therapeutic options in hospitals, leading to long-term hospitalizations, increases healthcare expenditure, and leads to higher mortality and morbidity rates [2]. AMR reduces the productivity of patients or their caregivers due to prolonged hospital stays. The annual increase of the extra healthcare expenditure was estimated to reach USD 0.22 trillion by 2030 in low AMR settings and USD 1.2 trillion in high AMR environments [3]. 

Several factors, including the common use of counterfeit antibiotics, self-medication in the communities, and unhygienic practices, have been speculated to contribute to the emergence and spread of multi-resistant bacteria in low- and middle-income countries (LMICs) [4,5]. Furthermore, the misuse of antibiotics, not only in human medicine but also in veterinary medicine, animal production, and agriculture, are the main factors that contribute to selecting and spreading MDR bacteria worldwide [6]. In LMICs, AMR is of particular concern due to the lack of resources in health care. Low socioeconomic status and poor hygiene in local communities are additional factors that exacerbate the AMR crisis [7]. Different contamination routes have been described, including human-to-human contamination, consumption of contaminated food, and direct contact with infected food-producing animals and pets [8,9]. Coordinated efforts described by the One Health concept are necessary to combat AMR in humans, animals, and the environment [10]. 

In 2024, a WHO report indicated carbapenem and 3rd-generation cephalosporin-resistant Enterobacterales, including *Escherichia coli* and *Klebsiella*, among the most critical MDR bacteria that represent a particular threat in healthcare worldwide [11]. Approximately 929,000 deaths directly attributable to AMR were caused by six pathogens, namely, *Escherichia coli*, *Staphylococcus aureus*, *Klebsiella pneumoniae*, *Streptococcus pneumoniae*, *Acinetobacter baumannii*, and *Pseudomonas aeruginosa*, in 2019 [1]. In recent decades, the emergence and spread of carbapenem-resistant Enterobacterales (CRE) have increased worldwide [12]. Carbapenems are last resort β-lactam antibiotics for the treatment of infections caused by MDR gram-negative bacteria. CREs either produce enzymes that can hydrolyze carbapenems (carbapenemase) or exhibit hyperproduction of AmpC β-lactamases or extended-spectrum β-lactamases (ESBL) combined with a loss of permeability of altered membrane [13]. The main carbapenemase classes according to Ambler’s classification are class A, *Klebsiella pneumoniae* carbapenemase (KPC); class B, metallo-β-lactamase (MBL) New Delhi metallo-β-lactamase (NDM), Verona integron-encoded metallo-β-lactamase (VIM) and Imipenemase (IMP); and class D OXA-48-like enzymes [14]. Carbapenemases can hydrolyze all β-lactams, causing serious public health concerns since β-lactams are widely used in hospitals to treat patients [15]. Unfortunately, recent studies in Burkina Faso and across Africa have reported the occurrence of CRE in hospitals and the community [16,17,18,19]. Carbapenem resistance genes, including *bla*_NDM_, were identified in isolates from environmental samples from South Africa, Tunisia, Egypt, and Nigeria [9]. 

In the environment, besides antibiotic-resistant bacteria, antibiotic residues can lead to the emergence and spread of AMR [7]. In the farm environment, vegetables can be contaminated by extended-spectrum β-lactamase-producing Enterobacterales (ESBL-E) through the use of improperly treated manure or contaminated irrigation water [20,21]. Agricultural soils serve as a natural reservoir of enteric pathogens, and animal manure used as fertilizer contains enteric pathogens that can persist in soils for months or even years, resulting in possible contamination of fresh produce [22]. Contaminated irrigation water has been identified as one of the common drivers of the occurrence of resistant bacteria in the agricultural environment [23,24]. Also, in Burkina Faso, irrigation water can originate from several sources, including wells, surface water, and wastewater from households and even hospitals [25,26]. ESBL-E can persist on the surface of plants or reach their interior and be transmitted to humans or animals [21,23]. Many studies have shown the presence of MDR Enterobacterales in fresh vegetables, such as lettuce and tomato [9,26]. Consumption of these contaminated raw vegetables can potentially negatively impact human health, as pathogenic agents can transfer their AMR genes to commensal bacteria that colonize the human gut [27]. The increased fecal carriage of the resistant bacteria may lead to a serious infection caused by MDR bacteria, which is difficult to treat, and poor hygienic conditions can spread the bacteria further into the environment [28,29]. The Ministry of Health in Burkina Faso recently reported an alarming trend in bacterial resistance to third-generation cephalosporins (β-lactams) within hospitals, up from 50% in 2018 to 60% in 2019 [30]. Recent studies in the country have highlighted the presence of ESBL-E in hospitals, communities, poultry farms, breeding animals, and lettuce and manure [31,32,33,34,35]. However, no study has specifically focused on ESBL-E and carbapenemase producers using a “One Health” approach in urban agriculture, although market gardens, i.e., commercial urban small-scale vegetable farms, are an important source of fresh produce for city dwellers and also provide an important source of income for numerous farmers. The purpose of this study was to assess the prevalence of extended-spectrum β-lactamase- and carbapenemase- producing *Escherichia coli* and *Klebsiella* spp. in market gardens in Ouagadougou, Burkina Faso, using a One Health approach.

## 2. Materials and Methods

### 2.1. Sites and Period of the Study

**Figure 1:**
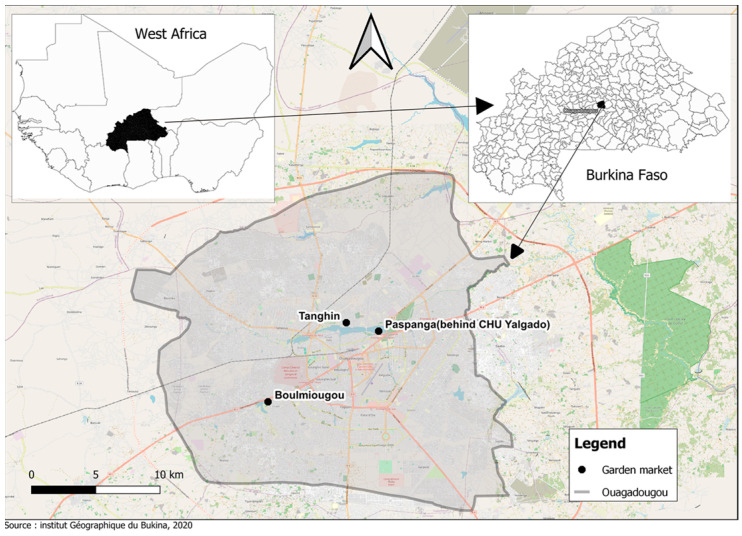
Map showing the sampling sites.

The samples were collected from three market garden sites in Ouagadougou from April 2021 to June 2022. Two sites were microbiologically polluted: the Paspanga gardening site is close to the largest hospital, Centre Hospitalier Universitaire Yalgado Ouédraogo, and near a channel that conducts municipal wastewater out of the city. The Tanghin gardening site is close to the medical center Hôpital Protestant Schiphra and next to a cattle market. On the contrary, the gardening site at the Boulmiougou Dam is located in a less polluted area far from health facilities (Figure 1). 

### 2.2. Sampling

A total of 356 samples, including lettuce (76), manure (62), soil (76), irrigation water (63), and human stools (79) samples, were collected from the three sampling sites. Approximately 50 to 100 g of lettuce leaves, 100 g of soil, or organic manure were aseptically collected in sterile bags. Irrigation water was collected into 500 mL sterile bottles. The gardeners were asked to provide their stool samples in sterile vials. All the samples were immediately placed in a cooler containing ice blocks and transported within two hours to the Unité génomique des pathogènes—One health/LaBESTA at Université Joseph KI-ZERBO for analysis.

### 2.3. Bacterial Isolation and Identification

Samples other than human stools were first enriched as follows: 10 g of soil or manure sample were mixed in 90 mL of buffered peptone water (BPW); 25 g of lettuce leaves were added into 225 mL of BPW; and 250 mL of irrigation water was filtered using a 0.45 µm filter membrane, which was placed into 9 mL of BPW. The samples in BPW were incubated at 37° C for 24 h. After incubation, 10 µL was plated onto selective CHROMagar ESBL agar plates (CHROMagar™ ESBL, Paris, France). Human stool samples were streaked directly onto the CHROMagar ESBL plates. A positive control was prepared for each sample by inoculating a non-selective cystine lactose electrolyte deficient (CLED) agar plate. All the inoculated plates were incubated at 37° C for 24 h. After incubation, the plates were inspected for different morphotypes of bacterial colonies according to the manufacturer’s instructions. Red or pink colonies were considered *E. coli*, whereas blue, green, or blue-green colonies were considered KESC (*Klebsiella*, *Enterobacter*, *Serratia*, and *Citrobacter*) group. One colony of each morphotype (*E. coli* or KESC group) per sample was purified using eosin-methylene blue agar (EMB). The purified isolates were transferred to Muller–Hinton agar and subsequently identified using biochemical tests, including glucose and lactose fermentation, gas and H_2_S production on Kligler iron agar (KIA), citrate utilization, mobility, and indole test. The verified ESBL-*Ec* and ESBL-*K* isolates were stored in 30% glycerol at −20 °C for further analysis.

### 2.4. Phenotypic Confirmation of ESBL Production

The double synergy test (DST) between a 3rd-generation cephalosporin (cefotaxime, C3G), a 4th-generation cephalosporin (cefepime, C4G), and amoxicillin + clavulanic acid (AMC) was used to confirm ESBL production [36]. The result was interpreted as positive when there was a visible synergy inhibition zone between C3G-AMC-C4G.

### 2.5. Antimicrobial Susceptibility Testing

Antimicrobial susceptibility testing was done using the disk diffusion method on Muller–Hinton (MH) agar [37]. Thirteen antibiotic agents commonly used in human and veterinary medicine were tested: amoxicillin + clavulanic acid (30 μg), cefoxitin (30 μg), cefotaxime (10 μg), cefepime (30 μg), meropenem (10 μg), gentamycin (10 μg), amikacin (30 μg), ciprofloxacin (5 μg), ofloxacin (5 μg), nalidixic acid (30 μg), tetracycline (30 μg), sulfamethoxazole+ trimethoprim (25 μg), chloramphenicol (30 μg), (Liofilchem, Italy). The results were interpreted following the recommendations of the American Clinical and Laboratory Standard Institute (CLSI) 2021 [38]. *Escherichia coli* ATCC 25922 was used for quality control of the antibiotic discs.

### 2.6. Detection of Carbapenemase Production

Isolates were first screened for putative carbapenemase production using meropenem disks. Isolates that exhibited a meropenem inhibition zone diameter of less than 22 mm were tested for carbapenemase production using the immunochromatographic test O.K.N.V.I. RESIST-5 (CORIS BioConcept, Gembloux, Belgium), according to the manufacturer’s instructions [36]. This immunochromatographic test can identify the five main carbapenemases: oxacillinase (OXA-48-like), *Klebsiella pneumoniae* carbapenemase (KPC), New Delhi metallo-β-lactamase (NDM), Verona integron-encoded metallo-β-lactamase (VIM), and imipenemase (IMP).

## 3. Results

### 3.1. Prevalence of ESBL-Ec and ESBL-K in Different Sample Types

Out of the 356 samples, 232 samples harbored at least one ESBL-producing *E. coli* (ESBL-*Ec*) and/or ESBL-producing *Klebsiella* spp. (ESBL-*K*) strain. Thus, the overall prevalence of ESBL-*Ec* and/or ESBL-*K* was 65.2%. A total of 274 strains were isolated, including 93 ESBL-*Ec* and 181 ESBL-*K*. The highest prevalence of ESBL-*Ec* was detected in human stools (53.2%), whereas the highest prevalence of ESBL-*K* was found in irrigation water, 68.3% (Table 1). 

### 3.2. Prevalence of ESBL-Ec and ESBL-K at Different Sites

**Figure 2:**
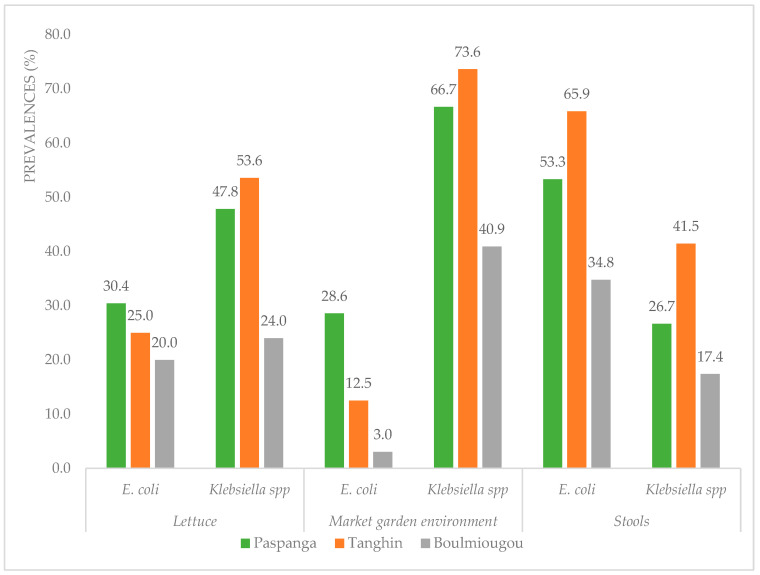
Prevalence of ESBL-*Ec* and ESBL-*K* at the sampling sites.

The samples from the Paspanga and Tanghin sites had a higher prevalence of both ESBL-*Ec* and ESBL-*K* in all sample types than the samples from the Boulmiougou sites. In lettuce, a high prevalence of ESBL-*K* was detected at Paspanga, 47.8%, and at Tanghin, 53.6% (Figure 2).

In the garden environment (soil, manure, and irrigation water), a high prevalence of ESBL-*K* was observed at Paspanga, 66.7%, and at Tanghin, 73.6%.

In human samples, the prevalence of ESBL-*Ec* was particularly high at Paspanga, 53.3%, and at Tanghin, 65.9%.

### 3.3. Antibiotic Resistance

The isolates showed high resistance against sulfamethoxazole + trimethoprim and tetracycline. ESBL-*Ec* and ESBL-*K* were most commonly resistant to sulfamethoxazole + trimethoprim 82.8% and 87.3% and tetracycline 86.0% and 89.0% (Table 2). Multidrug resistance (resistance to at least three different families of antibiotics) was detected in 74/93 (79.6%) ESBL-*Ec* and 158/181 (87.3%) ESBL-*K* isolates. Amikacin and meropenem (a carbapenem) remained most active against ESBL-*Ec* and ESBL-*K*.

### 3.4. Prevalence of AmpC-ESBL and Carbapenemase Producers at the Market Gardens Sites

Ten bacterial isolates (10/356) showing a cefoxitin inhibition zone diameter of <18 mm (2 ESBL-*Ec* and 8 ESBL-*K* isolates) were considered presumptive AmpC-producers. Four isolates resistant to meropenem (4 ESBL-*K*) were tested for carbapenemase production (OXA-48, KPC, NDM, VIM, and IMP). Two were positive for New Delhi metallo-*β*-lactamase (NDM) production. Carbapenemase-producing bacteria were detected in an irrigation water sample and a manure sample from the Paspanga site.

## 4. Discussion

This study described, for the first time, the occurrence and antimicrobial resistance of ESBL-producing *Escherichia coli* (ESBL*-Ec)* and *Klebsiella* spp. (ESBL-*K*) in urban agriculture in Burkina Faso. The sampling applied the One Health approach, which compared the prevalence in different possible sources of human infection with the prevalence in the farmers’ stools.

More than 65% of the 356 samples analyzed contained ESBL-*Ec* and/or ESBL-*K*. This high prevalence of ESBL-producing bacteria may be explained by the fact that β-lactams are commonly used antibiotics in human and animal healthcare [35,39]. Due to the lack of proper sanitation and wastewater treatment, bacteria resistant to β-lactams are released via human or animal feces or urine into the open runoff and drainage channels [7,40]. At our study sites, well water is the main source of irrigation, but some farmers use surface water or wastewater from the municipality wastewater channels. Furthermore, the gardening sites are situated in the open air, alongside water sources (wells, dams), without any protective measures against environmental contamination factors such as urban waste and animals. Contamination of fresh vegetables is known to occur from many sources, such as soil, farming equipment, animal manure, human handling, and post-harvest cleaning processes. However, irrigation water is considered to be the most important source of fresh produce contamination [41].

A high prevalence of ESBL-*Ec* and ESBL-*K* was observed at the sites in Paspanga and Tanghin, which are close to hospitals and a cattle market. In contrast, the Boulmiougou site, located in a less polluted area, far from health facilities, was less polluted. This was expected, as the hospitals and the cattle market release their waste and wastewater into the environment with little or no purification, leading to contamination of irrigation water and, consecutively, of soil, manure, and fresh produce [18,42,43]. 

Recent studies in Burkina Faso reported the presence of ESBL-*Ec* and ESBL-*K* in lettuce sold at the market to be 73.44% and 21.88%, respectively, and in manure samples, 43.5% and 18.5%, respectively [35,44]. In a Nigerian study, ESBL-*Ec* prevalence in agricultural soil was reported to be 68%, in manure 84%, in irrigation water 28%, and vegetables 24% [25]. In our study, the prevalence of ESBL-*Ec* in agricultural soil, manure, irrigation water, and lettuce varied between 10% and 25%, whereas the prevalence of ESBL-*K* was higher, between 43% and 68%. 

The application of organic manure on agricultural fields has previously been identified as a transmission route of MDR bacteria to humans, animals, and the environment [45]. The presence of antibiotic residues and pesticides discharged into the environment through manure or irrigation water can contribute to the development of antibiotic resistance in the environmental microbiome through selection pressure [7,46]. Consequently, consuming poorly decontaminated fresh market garden products may increase human fecal carriage of ESBL-*Ec* and ESBL-*K* [47,48]. This might contribute to the high prevalence of fecal carriage of ESBL-*Ec* and ESBL-*K* among gardeners in this study, 53.6% and 30.4%, respectively. However, in Burkina Faso, researchers reported similar prevalence rates also among healthy volunteers [31], while a study in Chad showed a high prevalence of 76% for ESBL-*Ec* and a low prevalence of 13% for ESBL-*K* [49]. The common intestinal carriage of the ESBL-E contributes to their prevalence in patients visiting in healthcare centers. Infections caused by ESBL-E lead to complications in therapeutic treatment, high hospitalization costs, prolonged hospital stays, and increased mortality and morbidity [50]. 

The ESBL isolates in this study were typically resistant to sulfamethoxazole-trimethoprim and tetracycline, as in a study on fresh vegetables conducted in South Africa [51]. We also detected rather common resistance to gentamycin, ciprofloxacin, ofloxacin, nalidixic acid, and chloramphenicol. The prevalence of MDR in ESBL*-Ec* was 79.6%, and in ESBL-*K*, 87.3%. The prevalence of MDR ESBL-*Ec* was lower than previously reported from environmental samples in Algeria (100%) but higher than in South Africa (33.3%) [52] or in a Swiss study, where vegetables imported from several LMICs were examined [53]. The low resistance rate against amikacin and meropenem both in ESBL-*Ec* and ESBL-*K* is consistent with recent studies conducted in Burkina Faso among ESBL-producing *E. coli* and *Klebsiella* isolates from wastewater and clinical samples [18,19]. 

In this study, two carbapenemase-producing ESBL-*K* isolates are reported, one from irrigation water and one from a manure sample from the Paspanga site. This site is located close to the hospital Centre Hospitalier Universitaire Yalgado Ouédraogo. Recent studies in Burkina Faso reported the presence of bacteria producing NDM in hospital patients and effluents near the same site [18,54]. This indicates that the hospital is a likely source of contamination of water, environment, and possibly even fresh produce. Studies conducted in Africa showed that NDM, OXA-48, and VIM were the most frequently detected carbapenemases in clinical, animal, and environmental samples [55].

This study focused on the phenotypic characterization of ESBL-*Ec* and ESBL-*K* strains in urban agriculture and an in-depth study by sequencing the resistance genes of these ESBL- and carbapenemase-producing bacteria would be needed to determine the linkage between the isolates from different sources and possibly reveal circulation of dangerous clones. The emergence of the carbapenemase-producing bacteria is of acute medical concern, as carriage of NDM genes is associated with high hydrolysis activity against all β-lactams and carbapenems except aztreonam [14,56]. Carbapenems are β-lactam antibiotics that should be used mainly as a last resort in the treatment of infections caused by gram-negative bacteria. The possible connection between the use of these antibiotics and the occurrence of the resistance genes against them in hospital wastewater and the environment outside the hospital requires further investigation. Good hygiene practices by consumers of fresh produce are necessary to prevent human contamination and limit the dissemination of resistant bacteria.

## 5. Conclusions

This study revealed a high prevalence of ESBL-*Ec* and ESBL-*K* in the market garden environment (irrigation water, soil, and manure), as well as in vegetable products (lettuce leaves) and gardeners’ stools in Ouagadougou. Furthermore, two isolates were found to produce NDM carbapenemase. These carbapenemase-producing bacteria were detected in irrigation water and in a manure sample from the Paspanga site, which is located near the largest hospital in Burkina Faso. Based on these findings, a multisectoral One Health approach should be implemented to understand and effectively control the spread of multidrug-resistant bacteria. Health authorities should oblige the health establishments to comply with the rules for the discharge of hospital liquid effluents into the environment. Regular monitoring is needed to enforce the effective management of hospital waste to limit the spread of resistant microorganisms into the environment. Also, in commercial gardening settings, monitoring contamination sources from humans (stools), animals (manure), and the environment (irrigation water and soil) should be applied by the municipality to protect the hygienic quality of fresh produce and the health of consumers.

## Figures and Tables

**Table 1:** Prevalence of ESBL-*Ec* and ESBL-*K* in different sample types.

Samples		N	ESBL-*Ec* (%)	ESBL-*K* (%)
Fresh produce	Lettuce	76	19 (25.0)	33 (43.4)
Market garden environment	Soil	76	8 (10.5)	47 (61.8)
	Manure	62	13 (21.0)	34 (54.8)
	Water	63	11 (17.5)	43 (68.3)
Human	Stools	79	42 (53.2)	24 (30.4)
Total		356	93 (26.1)	181 (50.8)

**Table 2:** Antibiotic resistance of the ESBL-*Ec* and ESBL-*K* isolates.

Antibiotic (μg)	Resistant ESBL-*Ec* (%)	Resistant ESBL-*K* (%)
Amoxicillin + clavulanic acid (30)	45/93 (48.4)	116/181 (64.1)
Meropenem (10)	0/93 (0.0)	4/181 (2.2)
Gentamycin (10)	17/93 (18.3)	40/181 (22.1)
Amikacin (30)	2/93 (2.2)	6/181 (3.3)
Ciprofloxacin (5)	32/93 (34.4)	46/181 (25.4)
Ofloxacin (5)	30/93 (32.3)	30/181 (16.6)
Nalidixic acid (30)	41/93 (44.1)	59/181 (32.6)
Tetracycline (30)	80/93 (86.0)	161/181 (89.0)
Sulfametoxazole+ trimethoprim (25)	77/93 (82.8)	158/181 (87.3)
Chloramphenicol (30)	5/93 (5.38)	42/181 (23.2)

## Data Availability

Data is contained within the article.
